# Feature Tracking: a novel method to analyze myocardial strain: Results from the CMR strain study in healthy volunteers

**DOI:** 10.1186/1532-429X-13-S1-P14

**Published:** 2011-02-02

**Authors:** Madhavi Kadiyala, Rena Toole, Kathleen Bertman, Simcha Pollack, Nathaniel Reichek

**Affiliations:** 1 Saint Francis Hospital, The Heart Center, Roslyn, NY, USA

## Objective

We sought to determine normal gender-specific strain values in healthy subjects using Feature Tracking (FT-MRI).

## Background

Feature tracking (FT-MRI) is a novel MRI based method to analyze myocardial strain that is fast, simple and has potential for clinical use. Similar to echocardiographic “speckle tracking” myocardial “features” can be tracked on routine steady state free precession or gradient echo images without the need for additional tagged imaging.

## Methods

Global and regional strains including circumferential (endocardial and epicardial Ecc), radial (Err) and longitudinal (Ell) strains were obtained with FT-MRI (Diogenes v Tomtec Systems) in 60 carefully screened normal subjects. Strains were derived from three long axis (four, three and two chamber) and three short axis views (basal, mid and apical ) after semi automated tracing of endocardial and epicardial borders. Strains were mapped to a 17 segment AHA model and regional strains in the left anterior descending (LAD), circumflex (LCX) and right coronary (RCA) territories derived.

## Results

**Figure 1:**
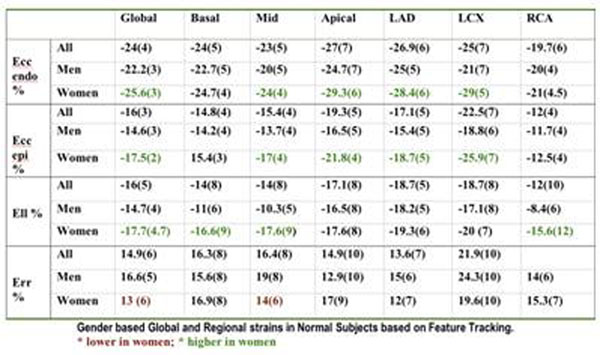


There were 29 men and 31 women with mean age 54(14) yrs. The peak global strains were : Ecc (endo) -24(4)%, Ecc (epi)-16(3)%( difference p <0.0001), Ell : -16(5)% and Err : 15(6)%. There was a significant mid to apical gradient in both Ecc (endo) and Ecc (epi) (Δ 4.4, p < 0.0001; Δ3.8, p <0.0001) and Ell (Δ 3, p =0.05), but no significant gradient was present in Err. The septum had the lowest Err (8.6(6)% ), while the lateral wall had the highest Err (23.9(9)%) . Err was highest in the LCX territory. Ecc, and Ell were lowest in the RCA territory. Ecc and Ell were generally higher in women than in men, while global Err was lower in women (fig). There was no significant relationship between the global strains and age, blood pressure, cholesterol levels, smoking history and family history of coronary disease. However the strains were significantly associated with height(r 0.5, p0.0002), weight (r 0.44, p 0.002), left ventricular end diastolic volume (r 0.57, p <0.0001) and ejection fraction (r -0.62, p <0.0001). Global Ecc (endocardial) correlated most closely with global ejection fraction (r=0.62, p<0.00001). Figure 1.

## Conclusions

FT-MRI is a novel MRI based method that provides consistent values for global and regional strains in three dimensions from conventional MRI images. There is significant variation in global and regional strains among men and women. There is significant regional and coronary territory heterogeneity of strain patterns.

